# Prevention of Mother-to-Child Transmission of HIV and Paediatric HIV Care and Treatment Monitoring: From Measuring Process to Impact and Elimination of Mother-to-Child Transmission of HIV

**DOI:** 10.1007/s10461-016-1670-9

**Published:** 2017-01-06

**Authors:** Priscilla Idele, Chika Hayashi, Tyler Porth, Awandha Mamahit, Mary Mahy

**Affiliations:** 1 United Nations Children’s Fund, Data and Analytics Section , 3 UN Plaza, New York, NY 10017 USA; 2 HIV Department, World Health Organization, Geneva, Switzerland; 3 Joint United Nations Programme on HIV/AIDS, Geneva, Switzerland

**Keywords:** HIV, AIDS, PMTCT monitoring, Paediatric HIV care and treatment, Global and national monitoring

## Abstract

Progress towards achievement of global targets for the prevention of mother-to-child transmission of HIV (PMTCT) and paediatric HIV care and treatment is an integral part of global and national HIV and AIDS responses. This paper documents the development of the global and national monitoring and reporting systems for PMTCT and paediatric HIV care and treatment programmes, achievements and remaining challenges. A review of the development of the monitoring and reporting process since 2002–2016 was conducted using existing published literature and taking into account changes in WHO HIV treatment guidelines, global HIV goals and targets, programmatic and methodological developments, and increased need for interagency partnerships, coordination and harmonization of global monitoring and reporting mechanisms. The number and type of indicators reported increased and evolved from monitoring of existence of national policies and guidelines, service delivery sites and trained health workers and coverage of PMTCT and paediatric HIV interventions to measuring outcomes and impact in reducing new HIV infections and AIDS related deaths, including efforts to validate elimination of mother-to-child transmission of HIV. These changes were required to mirror changes in WHO and national PMTCT and HIV treatment guidelines. The number of countries reporting PMTCT coverage increased from 53 in 2003 to over 130 in 2015. National monitoring processes have also expanded in scope and the capacity to report on disaggregated data by type of ARV regimen and for paediatric HIV care and treatment has increased. Monitoring of PMTCT and paediatric HIV programmes has contributed a rich body of evidence that helped monitor how quickly countries were adopting and implementing the latest WHO HIV treatment guidelines for pregnant and breastfeeding women and children. The reported data and experiences were instrumental in shaping global policies, national programmes, and investment choices.

## Background and Objectives

In 2000, member states committed to Millennium Development Goals (MDG) 4, 5, and 6 on health for women and children: reduce child mortality, improve maternal health and halt and begin to reverse the spread of HIV and AIDS by 2015 [1]. At the United Nations General Assembly Special Session on HIV and AIDS (UNGASS) in 2001, member states further committed to reducing the proportion of infants infected by HIV by 20% by 2005 and 50% by 2010, and ensuring 80% of pregnant women accessing antenatal care should receive information, preventive services and treatment to reduce mother-to-child transmission of HIV, voluntary and confidential counselling and testing, access to treatment, especially antiretroviral therapy, and where appropriate, breastmilk substitutes and continuum of care [2]. The High Level Global Partners Forum in Abuja, Nigeria in 2005 convened by the Inter-Agency Task Team on Preventing HIV Infection in Women, Mothers, and their Children (IATT), together with governments, donors and implementing partners resulted in a Call to Action for the Elimination of HIV Infection in Infants and Children [3]. In the same year, the United Nations International Children’s Fund (UNICEF) and the Joint United Nations Programme on HIV/AIDS (UNAIDS) launched the global *Unite for Children Unite against AIDS* campaign to support universal access to treatment and address the impact of HIV and AIDS on children [4].

Since 2001, the HIV/AIDS monitoring and evaluation reference group (MERG), that brings together UN agencies, donors, implementing partners, government and civil society, played a critical role as a coordinating body for all HIV monitoring and a common and harmonized M&E framework for global and national reporting and monitoring of progress. More recently, the global community committed to the goal of eliminating paediatric HIV infections by 2015 [5]. The *Global Plan Towards the Elimination of New HIV Infections Among Children by 2015 and Keeping Their Mothers Alive* adopted in 2011 *(The Global Plan)* includes a monitoring and evaluation (M&E) framework with specific targets, indicators and baselines against which progress is assessed [6]. At the same time, the 2011 *Political Declaration on HIV and AIDS: Intensifying Our Efforts to Eliminate HIV and AIDS* included a target of elimination of mother to child transmission of HIV [7].

Since 2002, there has been unprecedented investment and efforts in developing harmonized global and national monitoring systems, reporting processes, and coordination mechanisms among UN organizations and key partners. The goal of this investment was to create data to inform policy, programming, track progress and ensure accountability of country results. This paper documents the development of monitoring and reporting systems for PMTCT and paediatric HIV care and treatment programmes. The paper also highlights some of the key achievements towards the global targets, outlines the challenges in data availability and quality, and proposes some areas for further strengthening and development in the monitoring of the HIV response in PMTCT and paediatric HIV care and treatment.

## Methods

A document review of the MDGs, the UNGASS Declaration of Commitment, The Global Plan, the 2011 Political Declaration on HIV/AIDS, as well as, reports of high level meetings and IATT consultations was conducted to contextualize the basis and process of the establishment of the PMTCT and paediatric HIV care and treatment monitoring systems. Published M&E guidance on PMTCT and paediatric HIV care and treatment was reviewed to assess the evolving nature of the monitoring and evaluation system since 2002–2016, with specific emphasis on types of indicators and coordination mechanisms. Annual reports of comparable data on a set of core indicators by countries to UNAIDS, UNICEF and the World Health Organization (WHO), were also reviewed to assess progress towards achieving global and national goals and targets, as well as, data availability and quality. A review of published guidelines and reports on methodological and programmatic developments was done to assess advances in measuring PMTCT and paediatric HIV care and treatment programme outcomes and impact.

## Results

### Global Coordination of PMTCT and Paediatric HIV Monitoring

Under the leadership of WHO and UNICEF, the IATT on PMTCT M&E formed in 2005 has played a major role in reviewing methodologies and technical issues and providing guidelines related to monitoring of PMTCT and paediatric HIV care and treatment [8]. National and international experts and leading academic scholars have been involved in developing the internationally agreed definitions, classification, standards and recommendations for PMTCT and paediatric indicators. This work is undertaken through thematic sub-groups established within the IATT mechanism that brings together more than 30 specialized organizations and numerous experts.

WHO, UNICEF and UNAIDS ensure the coherence among existing global initiatives in the collection and compilation of HIV and AIDS data, including for PMTCT and paediatric HIV, ensure the harmonization of the M&E standards and methods for indicators and data collection, and the coordination of capacity building and technical assistance activities in countries for the production of high quality data and use at both global and country levels. The three United Nations (UN) organizations collaborate very closely with other key partners including the President’s Emergency Plan for AIDS Relief (PEPFAR) and the Global Fund for TB and Malaria (GFATM) and ensure harmonization of indicators, data, and processes.

Table 1 summarizes the development of the process of monitoring PMTCT and paediatric HIV care and treatment programmes at global level. The UN agencies have established a joint monitoring process for assessing progress towards global and national targets on PMTCT and paediatric HIV in line with various global commitments. UNICEF led the initial pilot of PMTCT programmes in 2002, and established a monitoring system, that excluded paediatric HIV. In 2003 UNAIDS and partners called on countries to submit UNGASS reports that included the PMTCT and paediatric indicators [9]. Between 2004 and 2008, WHO and UNICEF jointly requested the same data directly from their country offices and published PMTCT and paediatric HIV report cards and universal access reports on the health sector response. In 2009 the three UN agencies merged the monitoring processes into one spreadsheet-based reporting tool within the UNGASS framework, and in 2011, this was renamed the Global AIDS Response Progress Reporting (GARPR) system, which is now an online platform. Since then, the HIV response reporting is a joint mechanism of UNAIDS, WHO and UNICEF, aimed to reduce reporting burden on countries, harmonization of monitoring efforts and greater comparability and use of data across countries, regions, and across the three UN agencies.

**Table 1:** Development of the process of monitoring PMTCT and paediatric HIV care and treatment programmes

2000–2004	2005–2008	2009–2010	2011–2013	2014–2016
2000 UNAIDS M&E guide for national programmes	2005-2008 UNICEF & WHO jointly facilitate country reporting and publications on behalf of the IATT	WHO, UNICEF, & UNAIDS joint reporting and publications under the universal access goals and on behalf of IATT	2011-2012 WHO, UNICEF & UNAIDS transition to a joint reporting and merged with UNGASS using an online excel-based reporting tool	2014 UNAIDS, WHO and UNICEF transition to the Global AIDS Response Progress Reporting (GARPR) online tool and publish the Global Plan progress report
2002 UNICEF initiates monitoring and reporting for PMTCT pilot countries	2006 WHO HIV treatment guidelines	2009 UNAIDS Monitoring the Declaration of Commitment on HIV/AIDS: Guidelines on construction of core indicators	2011 UNAIDS Global Plan M&E Framework;	2014 Option B + M&E Framework
2003 WHO PMTCT M&E guide	2007 UNAIDS Monitoring the Declaration of Commitment on HIV/AIDS: Guidelines on construction of core indicators	2010 WHO HIV treatment guidelines	2012 & 2013 UNAIDS Global Plan Progress reports	2015 WHO Consolidated Strategic Information Guidelines
2003, 2005 UNAIDS Monitoring the Declaration of Commitment on HIV/AIDS: Guidelines on construction of core indicators		2010 WHO PMTCT and paediatric HIV M&E guide for national programmes	2013 WHO HIV treatment guidelines	2014, 2015 & 2016 Global Plan progress reports
		2011 WHO/UNICEF/UNAIDS Universal Access report		

United Nations organizations also collaborate on reviewing, validating and analyzing country reported data with other key partners, mainly PEPFAR and the GFATM. The data are reviewed for consistency, gaps and any unusual spikes or drops in coverage levels for key indicators. In addition the data are triangulated with data submitted by countries on national HIV estimates files which require data on the number of pregnant women receiving antiretroviral medicine to prevent MTCT and children receiving antiretroviral therapy [10]. The HIV estimates are generated annually by country teams and include estimates of the numbers of adults and children living with HIV, new HIV infections, AIDS-related deaths, as well as, mother-to-child transmission (MTCT) rates and the need for PMTCT. These estimates are currently used in the calculation of 8 of the 10 Global Plan indicators. These data are summarized and published in global reports by UN organizations [11].

### Evolution of PMTCT and Paediatric HIV Monitoring and Evaluation Recommendations

Between 2000 and 2015, recommendations for routine PMTCT monitoring have evolved in scope and content alongside WHO PMTCT and HIV treatment guidelines and national programmes (Table 2). National PMTCT monitoring has shifted from counting the number of pregnant women tested and receiving a single dose of nevirapine, to monitoring the type of ARV regimen received during the antenatal, delivery and breastfeeding periods, tracking follow-up care in children including early infant diagnosis and paediatric treatment coverage disaggregated by key age groups, and to the routine collection of final HIV outcome status. Impact assessment of PMTCT is emphasized particularly in high prevalence countries. More recently, and as countries move toward significant reductions in MTCT of HIV, WHO has led the process of developing guidance for validation of the elimination of MTCT as a public health problem, to enable countries assess progress towards reaching the targets set in the Global Plan [12]. This expansion and improvement in national M&E systems has led to further developments including the review and use of data sub-nationally often facilitated by electronic district health information systems (DHIS) and the use of positivity rates from routine programme data on HIV testing at ANC for HIV surveillance purposes.

**Table 2:** Evolution of key PMTCT and paediatric HIV care and treatment indicators for monitoring and evaluation of the global and national responses

Phases	Key indicators
Phase 1: 2000–2005	Focus on process monitoring Existence of guidelines on PMTCT; availability of trained health workers; number of health facilities providing PMTCT services; number of pregnant women tested for HIV and receiving their results; and number of HIV positive pregnant women receiving antiretroviral prophylaxis (ARVs) for PMTCT
Phase 2: 2006–2010	Emphasis on follow up and care of HIV positive pregnant women and postpartum mothers and their children For mothers—use of more efficacious prophylactic regimens, assessment for ART eligibility; ART of eligible HIV-infected pregnant and breastfeeding women; family planning; disaggregation of ARVs for PMTCT by type of regimen For children: cotrimoxazole prophylaxis, early diagnosis for HIV, ART for eligible; and exclusive breastfeeding
Phase 3: 2011–2013	Monitoring of lifelong ART to all HIV positive pregnant and breastfeeding women Addition of new paediatric HIV infections; MTCT rate; AIDS-related maternal and child mortality; disaggregation by pregnancy status, and by timing of initiation of ARVs
Phase 4: 2014–2015	Monitoring of HIV cascade, including impact and EMTCT targets Many countries were able to report PMTCT data by sub-national areas and on a 6-monthly basis. For ART a number of countries were able to report by more specific age groups Additon of routine reporting of PMTCT outcomes, retention on ART, viral suppression for those on treatment; 50 or fewer new paediatric HIV infections per 100,000 live births and a transmission rate of either 5% or less in breastfeeding populations or 2% or less in non-breastfeeding populations

In the early 2000s (first phase) when PMTCT programmes were just starting in many countries, global PMTCT indicators focused on monitoring inputs, such as the existence of guidelines on PMTCT, the number of trained health workers, the number of health facilities providing services, the number of pregnant women tested for HIV and receiving their results, and the number receiving ARV prophylaxis. The indicators are summarized in the 2004 guidelines for monitoring and evaluation of PMTCT programmes developed by WHO and partners [13]. The collection and reporting on these indicators was also included in the UNAIDS guidelines on Monitoring the Declaration of Commitment on HIV/AIDS of 2003 and 2005, which helped promote their use at country level [14, 15].

In the mid-2000s (second phase), as PMTCT interventions were scaling up, revised WHO guidelines in 2006 and 2010 called for an update of existing indicators, disaggregation, and development of new indicators. The focus shifted to monitoring follow-up and care of HIV positive pregnant and breastfeeding women, the use of more efficacious prophylactic regimens, HIV treatment of eligible HIV-infected pregnant and breastfeeding women, and unmet need for family planning among women living with HIV. Similarly, indicators for follow up and care of infants born to HIV positive mothers—cotrimoxazole prophylaxis, early diagnosis for HIV, access to paediatric ART and care, and exclusive breastfeeding—were also developed. In addition, the issue of potential double-counting was raised and explicitly addressed for the first time, calling for countries to review and develop mechanisms to minimize double counting and ensure reliable and consistent national data on multiple interventions across various service delivery platforms. Similarly, the UNAIDS guidelines on Monitoring the Declaration of Commitment on HIV/AIDS of 2007 and 2009 were updated to include the revised indicators [16, 17].

Aggregation of monthly or quarterly cross-sectional data often results in double-counting of women who access services in multiple facilities or multiple times at the same facility which could inflate national statistics. Retrospective cohort-based reporting was suggested as an alternative to aggregation of purely cross-sectional data. An important milestone during this phase was the recognition of the need to establish systems that can monitor linkages across service delivery clinics and sites. The interlinked patient monitoring tools for HIV care and ART, which outline a minimum data set and accompanying generic tools for data collection and reporting, were developed for country adaptation [18]. The patient monitoring tools aimed to integrate PMTCT and paediatric HIV interventions within maternal and child health (MCH) service provision, and support and monitor the provision of tuberculosis (TB) prophylaxis and screening and TB-ART co-treatment within HIV services.

The third phase (2011–2013) commenced with the launch of the Global Plan which set the ambitious goal of eliminating mother-to-child transmission by 2015. Recognizing the importance of assessing the impact of PMTCT programmes, the Global Plan M&E Framework included four impact indicators—HIV incidence among children aged 0–14 years, mother-to-child transmission rate, AIDS-related maternal deaths and child deaths for children under five. The revised 2013 WHO *Consolidated Guidelines on the Use of Antiretroviral Drugs for Treating and Preventing HIV Infection* recommended the initiation of antiretroviral therapy (ART) for all pregnant and breastfeeding women with HIV and, in many settings, continuation on ART for life (known as Option B+). This called for further refinement of indicators to align with the new recommendations, particularly retention of HIV positive mothers and HIV-exposed and infected children in ART care and treatment to monitor quality of care and the impact of ART on MTCT. The guidelines also re-emphasized the need to disaggregate coverage of ARVs for PMTCT by type of regimen but also to monitor ART retention by pregnancy status to better assess quality of care, country progress in adoption of more efficacious regimens, and the impact on MTCT rates. Consequently, it became inevitable to ensure PMTCT monitoring is aligned with ART monitoring, and with maternal, new born and child health services.

In the fourth phase (2014–2015), countries started to move toward measuring the Global Plan goal of eliminating new paediatric HIV infections by 2015. It became imperative to revise or develop indicators and guidelines for monitoring retention, assessing impact, but also validating country progress. In 2014, the IATT PMTCT M&E working group developed guidance for operationalizing M&E for lifelong ART for pregnant and breastfeeding women and their infants and aligned with the 2013 WHO HIV treatment guidelines [19]. The IATT PMTCT M&E guidelines recommend indicators for routine and enhanced monitoring and also for evaluating PMTCT programmes particularly in the early stages of rolling out lifelong ART for all pregnant and breastfeeding women, which is also aligned with the WHO 2015 consolidated strategic information (SI) guidelines for HIV in the health sector [20]. The WHO 2015 SI guide brought together all of the separate health related HIV M&E guidelines into one, with emphasis on the HIV cascade and linkages across multiple services (prevention, diagnosis, care, treatment, quality and impact) for all population groups, including for pregnant women, children and adolescents. WHO, in collaboration with UNICEF, UNAIDS, United Nations Population Fund (UNFPA) and other partners developed guidance on criteria and process for validating EMTCT and syphilis [21]. The guidance recommends two impact indicators for validating elimination of mother-to-child transmission (EMTCT) of HIV—50 or fewer new paediatric HIV infections per 100,000 live births and a final transmission rate of either 5% or less in breastfeeding populations or 2% or less in non-breastfeeding populations.

**Fig. 1:**
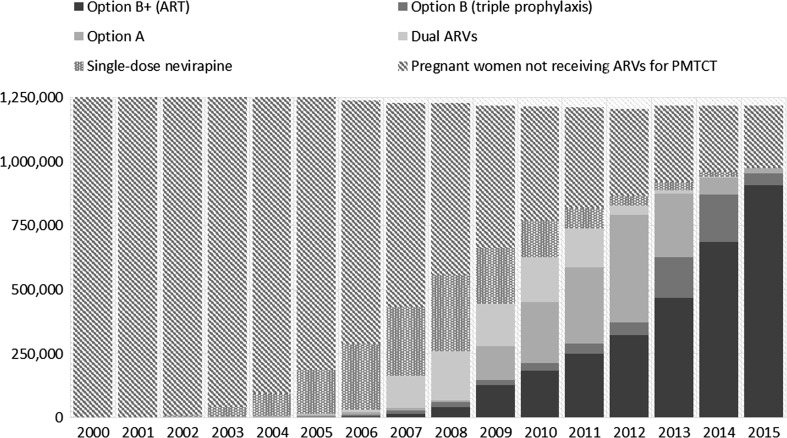
Distribution of the number of pregnant women living with HIV receiving antiretroviral medicines for PMTCT by regimen, 21 sub-Saharan African Global Plan countries, 2000–2015. *Source* UNAIDS/UNICEF/WHO Global AIDS Response Progress Reporting database, 2016

Disaggregation of ARVs by type of regimen has enabled countries to track progress on the adoption of more efficacious regimens, and assess the impact of regimen choice on their MTCT rates. The 2013 WHO recommendation for lifelong ART for PMTCT led to the rapid transition in the type of HIV treatment regimens used by countries. In 2005, 93% of pregnant women on ARVs for PMTCT in the 21 Global Plan priority countries were receiving single-dose nevirapine, 1% were receiving ART and only 6% were receiving other “ effective regimens“. By 2015, the pattern had reversed—with 92.8% receiving ART and 6.9% other effective regimens for PMTCT (Fig. 1) [22].

### Compiling Data and Reporting on the Progress Achieved Towards the PMTCT and Paediatric HIV Care and Treatment Targets

At the UN General Assembly Special Session on HIV countries committed to report on their progress toward reversing and halting the HIV epidemic. UNAIDS and co-sponsors were given the mandate to support countries to report on progress toward the UNGASS targets. The UNGASS declaration included targets to reduce transmission of HIV to children by 20% in 2005, and by 50% in 2010. The UNGASS monitoring framework identified the indicators used to measure the UNGASS targets and included two indicators related to PMTCT—the proportion of women living with HIV receiving antiretroviral medicines to prevent transmission of HIV to their children and the proportion of children born to women living with HIV infected with HIV (modelled). Paediatric ART access was captured by disaggregating the HIV treatment coverage indicator by adults and children (under 15 years and over 15 years).

**Fig. 2:**
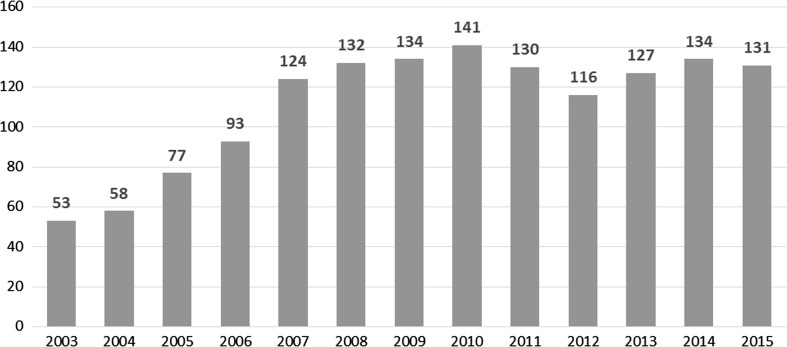
Number of countries reporting on coverage of ARVs for PMTCT, 2003–2015. *Source* UNICEF, PMTCT progress reporting, 2003–2006; WHO/UNICEF Universal Access Progress Reporting, 2007–2010; UNAIDS/UNICEF/WHO Global AIDS Response Progress Reporting databases, 2011–2015

By 2015 the number of countries reporting on PMTCT coverage more than doubled since the first UNGASS reports submitted in 2003. In 2003 only 53 countries reported data on the number of women receiving ARVs for PMTCT through national UNGASS reports or directly to UNICEF. The number of countries reporting dramatically increased to 124 countries in 2007 and by 2015, over 130 countries reported the number of women receiving ARVs (Fig. 2) [23]. In the 2003 data, half [27] of the 53 countries reporting stated that either 0 or <1% of women were receiving ARVs for PMTCT, suggesting very low coverage but also very weak systems for compiling these data. In 2015, only one of the 131 countries reporting stated that no pregnant women were receiving ARVs.

**Fig. 3:**
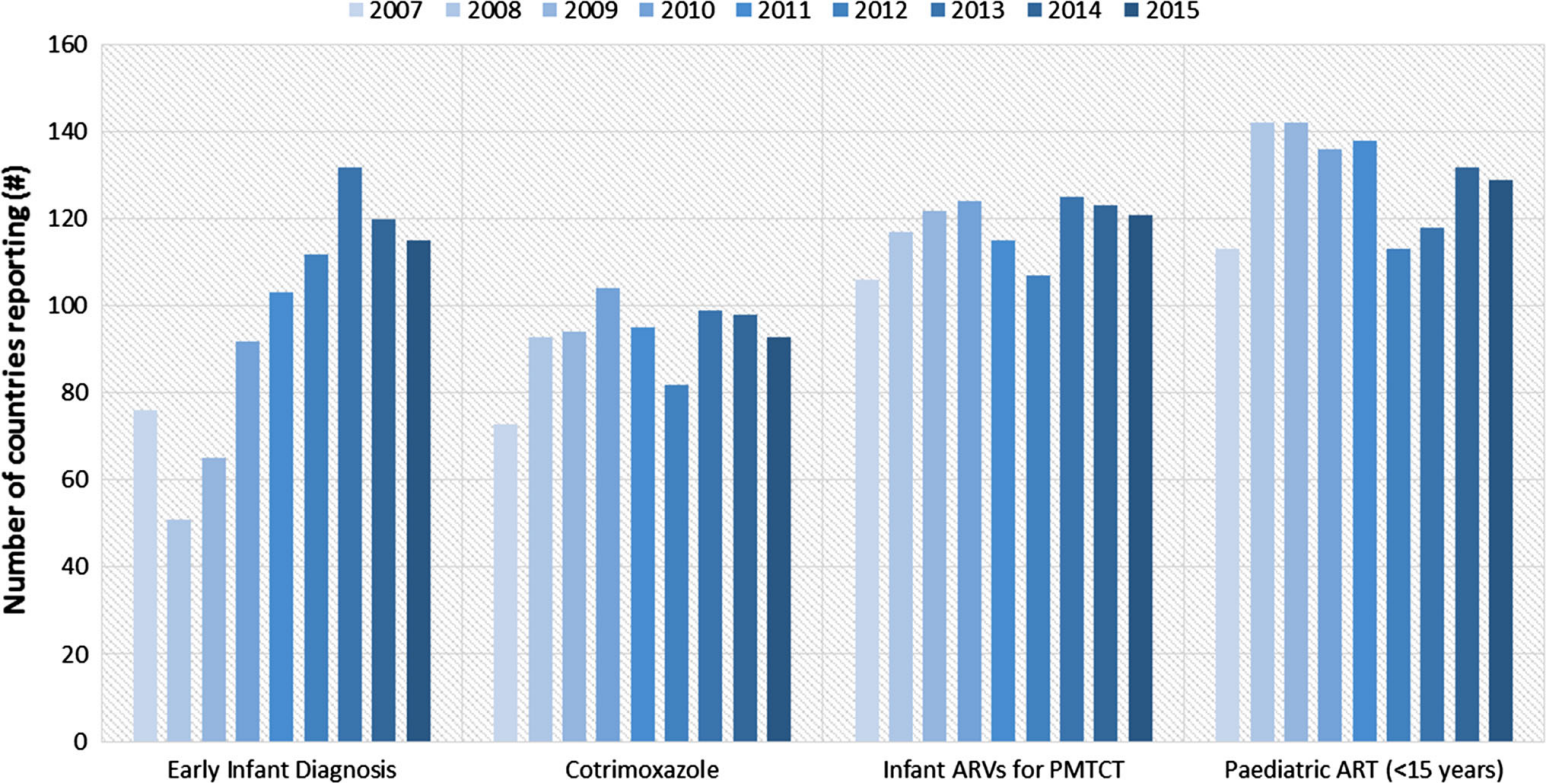
Number of countries reporting on paediatric HIV care and treatment indicators, 2007–2015. *Source* WHO/UNICEF Universal Access Progress Reporting, 2007–2010; UNAIDS/UNICEF/WHO Global AIDS Response Progress Reporting databases, 2011–2015

Overall, there has been a dramatic increase in availability of paediatric HIV care and treatment data across the key indicators since 2005. While the numbers of countries reporting ranged between 50 and 70 in earlier years and across the four key indicators on early HIV testing, cotrimoxazole prophlaxis, infant ARVs and paediatric ART, these rose to over 100 in 2011. About 130 countries reported on paediatric ART in 2015 (Fig. 3)

The remaining challenge is in ensuring availability of quality and complete data on all key interventions and outcomes. Few countries are able to systematically collect and report on complete reliable information on early infant diagnosis and more granular age disaggregated ART data for children. Most national monitoring systems have not been designed to report such data to the central level, even though these data may be available at the health facility level. Estimates for paediatric ART coverage were not published by UNAIDS in the early reports because of the challenges in estimating the number of children in need of ART. In 2010 the data were limited to countries with generalized epidemics with fewer estimation challenges. In 2015, estimating the number of children living with HIV and needing ART remains challenging especially in low level epidemics. A total of 54 countries were able to report on paediatric ART coverage, while 129 countries were able to report on the number of children receiving ART.

Since 2011 Progress Reports have been published to track progress toward the Global Plan. The impact indicators selected for monitoring the Global Plan—new HIV infections among children 0–14 years and MTCT rate—were highly reliant on models with little emphasis on developing routine monitoring systems to directly measure the impact of PMTCT programmes. Availability of programme coverage data to inform the modelled estimates, and in some countries sub-national estimates, provides insight on where the greatest gap is and which interventions are still lagging behind.

**Fig. 4:**
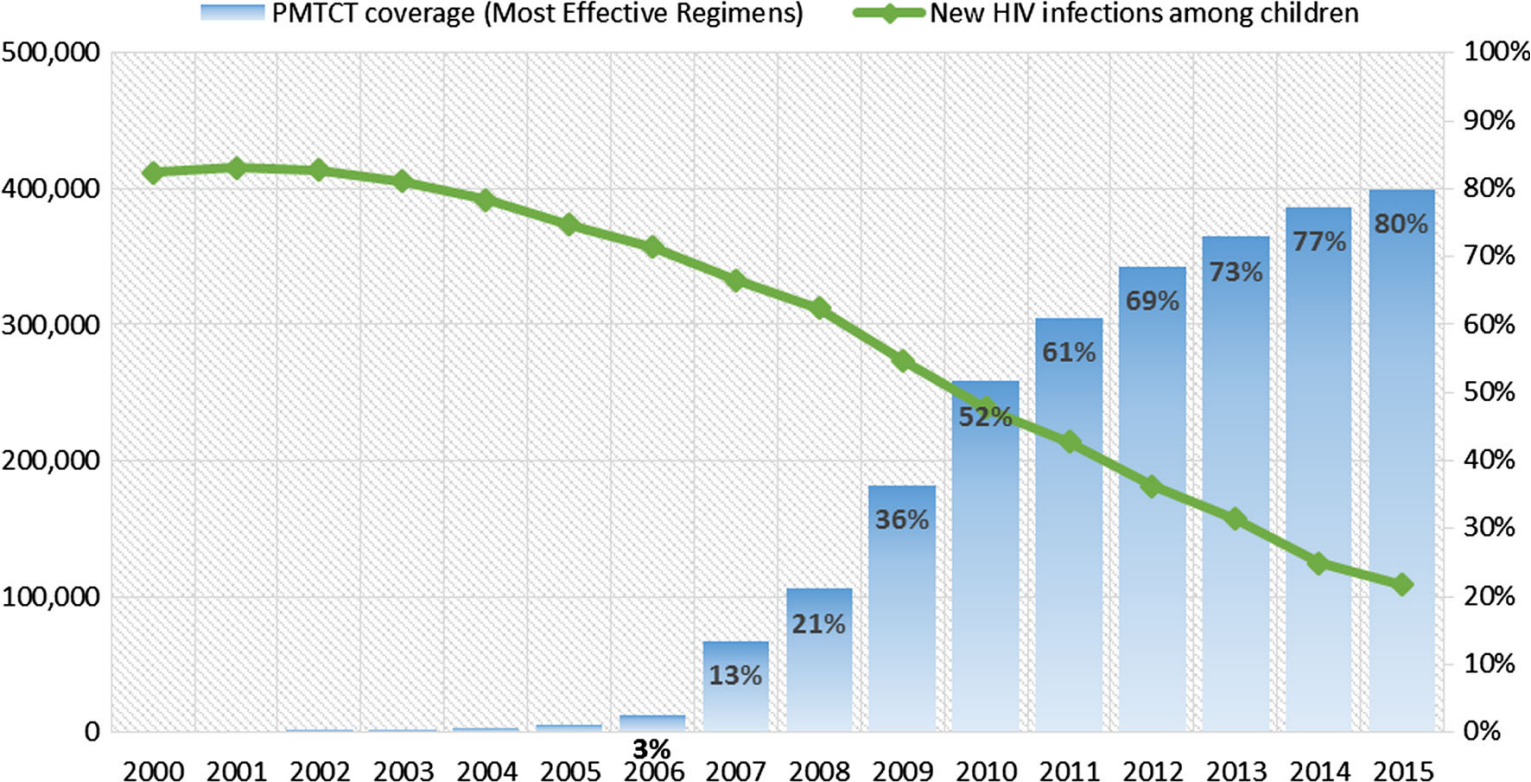
Trends in percentage of pregnant women living with HIV receiving effective antiretroviral medicines for PMTCT and new HIV infections among children 0–14, 21 sub-Saharan African Global Plan countries, 2000–2015. *Source* UNICEF, PMTCT progress reporting, 2002–2006; WHO/UNICEF Universal Access Progress Reporting, 2007–2010; UNAIDS/UNICEF/WHO Global AIDS Response Progress Reporting databases, 2011–2015; UNAIDS 2016 estimates, July 2016

The ambitious Global Plan targets coupled with global and country political commitments and concerted efforts has led to remarkable achievements. Globally, about 70% fewer children were newly infected with HIV in 2015 than in 2000 [24]. The dramatic scale up of HIV treatment among pregnant women living with HIV has translated into similar reductions in new infections among children 0–14 years in sub-Saharan Africa (Fig. 4). The rate of decline in new HIV infections in this group of children has accelerated in recent years, in line with the expansion of maternal ARV coverage in that region. Fewer HIV infections among children has also meant fewer AIDS-related child deaths. Since 2000, AIDS-related mortality among children under 5 years has fallen by approximately 70% globally, driven partly by reductions of 80% or more in 12 of the 21 Global Plan priority countries in sub-Saharan Africa during the same period [25].

**Fig. 5:**
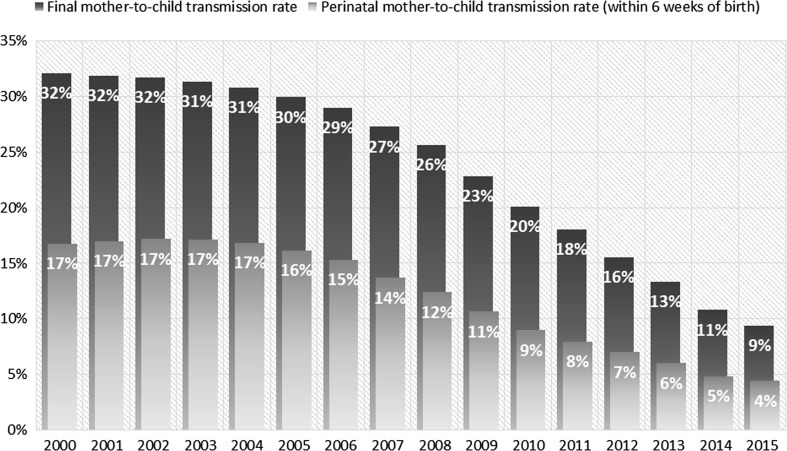
Estimated percentage of infants born to pregnant women living with HIV who are vertically infected with HIV (mother-to-child transmission rate), sub-Saharan Africa, 2000–2015. *Source* UNAIDS 2016 estimates, July 2016

As the monitoring data have improved so too have the models that are based on those data. The modelled estimates are generated based on specific assumptions of the demographic and HIV epidemiological trends and patterns among women of reproductive age as well as coverage of PMTCT services [26]. The Global Plan M&E framework has led to more in-depth analysis for the 21 sub-Saharan African countries prioritized under the Global Plan and what aspects of the MTCT response will have the largest impact on reducing new child infections and improving the well-being of mothers. Modelled data has been useful in highlighting the importance of retention, adherence, and follow up care of mothers and their children after delivery to minimize the risk of HIV transmission during the breastfeeding period. While there has been remarkable success in reducing new HIV infections among children during pregnancy and delivery, the mother-to-child transmission that is still occurring is probably largely during the postnatal risk period (Fig. 5). In 2000, MTCT rates in sub-Saharan Africa were estimated to be 17% in the perinatal period and 32% during the combined perinatal and postnatal period. By 2015, the estimated perinatal transmission rate was 4%, reaching the Global Plan target of 5%, while the final transmission rate was 9% (Fig. 5). Though there were markedly fewer infections overall in 2015, the infections are still occurring in about equal numbers during both perinatal and postnatal periods [27].

**Fig. 6:**
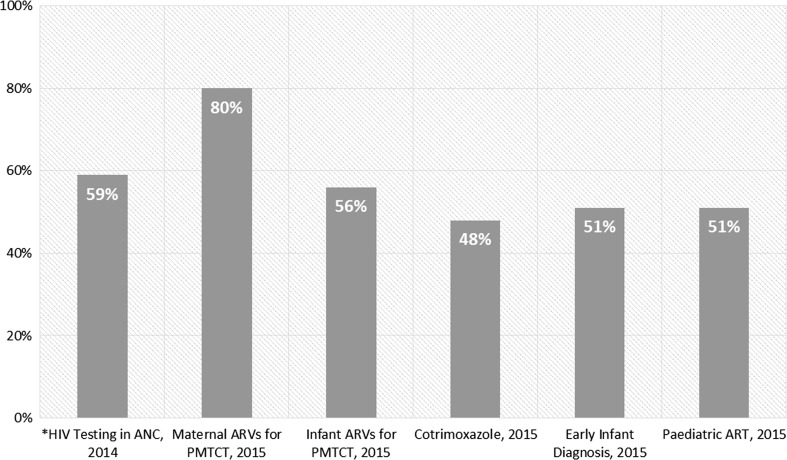
Coverage of key interventions for preventing mother-to-child transmission of HIV and for paediatric care and treatment among the 21 Global Plan priority countries in Africa, 2015. *Data on HIV Testing in ANC are for 2014. *Source* UNAIDS/UNICEF/WHO Global AIDS Response Progress Reporting, 2016

In 2014 only three Global Plan countries (Rwanda, South Africa and Zimbabwe) had conducted population level impact studies of their PMTCT programmes. The main challenge to measuring the population impact is tracking the children after delivery to the end of breastfeeding to determine their final HIV and survival status. Many countries have not developed routine systems to longitudinally monitor children who are born to HIV positive mothers. Overall, coverage for all of the relevant interventions is low among children (Fig. 6). In 2015, only 51% of the HIV exposed children in the 21 Global Plan priority countries in Africa had a virological test for HIV within the first two months of life and only 51% of those living with HIV received ART compared to 74% of their mothers (Fig. 6) [28].

## Discussion and Conclusions

The PMTCT and paediatric monitoring framework has strengthened information systems and fostered the use of data to improve programmes and ensure accountability by national governments and international organizations. Starting with fragmented global monitoring systems in the early 2000s, UNAIDS, WHO and UNICEF led the process of creating a coordinated and harmonized effort for HIV monitoring and reporting, including for PMTCT and paediatric HIV care and treatment. This resulted in reduced reporting burden on countries, created country ownership and accountability, and strengthened partnerships at both global and national levels, and brought coherence and harmonization in indicator definitions, guidelines, capacity building and technical support for M&E. The GFATM and PEPFAR key global and national monitoring indicators are now also harmonized with those of the UN organizations. While global monitoring and reporting among UN agencies has been harmonized, parallel reporting mechanisms and different timelines exist for PEPFAR and the GFATM.

The process of developing PMTCT and paediatric HIV care and treatment indicators and guidance has been inclusive and involved various organizations—UN organizations, multilaterals and bilaterals such as GFATM, United States Agency for International Development (USAID), Centers for Disease Control (CDC) and PEPFAR, international non-governmental organizations (NGOs), government representatives, civil society organizations (CSOs), people living with HIV and academia. The data collected jointly by UNAIDS, WHO and UNICEF are publicly available online on www.aidsinfo.unaids.org and also published in key reports, thus encouraging transparency and accountability. Many countries do not allow public access to their data, thus limiting analysis and use. Open and easily accessible data should be promoted to ensure government transparency and accountability and use of data for decision-making by government, citizens and other partners.

Monitoring of PMTCT and paediatric HIV programmes has also contributed to a rich body of evidence that has informed methodological and modelling processes. They have helped track the uptake of HIV treatment guidelines for pregnant and breastfeeding women, such as from less efficacious antiretrovirals (ARVs)—single dose nevirapine—to more efficacious simple to use lifelong combination ART of one pill a day, and HIV treatment for all children less than 5 years.

While availability of data on key indicators has dramatically increased, data quality for some of the indicators remains weak in a number of countries. Data incompleteness and inconsistencies in the values reported across indicators and time points are common. It is also difficult for countries to keep pace with frequent changes in WHO HIV treatment guidelines which may require revision of national monitoring systems and indicators every few years. The increased call for disaggregated and sub-national data is also making it difficult for countries to report data in the format that is required. In many countries ART data are only available in two broad age groups—under 15 years and over 15 years—making it difficult to assess progress in younger children and among adolescents. Reporting on ARVs for PMTCT regimens remains challenging since different regimens might be available in the same country, while patient registers do not allow for new regimen disaggregations. Similarly, the indicator on early infant diagnosis is often not reported accurately as the majority of children are tested beyond two months of birth, and even when the tests are conducted within two months, the average turnaround time for returning HIV test results is long and delays timely initiation of ART for those that need it. Countries will need support to strengthen the generation of relevant disaggregated data that can meaningfully inform targeting of limited resources to where there are most needed.

Currently, systems to monitor coverage indicators are well developed. However, few countries have established routine programme systems for monitoring the impact of PMTCT and paediatric HIV care and treatment programmes. Going forward, resources need to be mobilized and focused on developing robust routine monitoring systems to monitor new HIV infections and MTCT rates to the end of the breastfeeding period, including with maternal and child survival outcomes. Monitoring ART retention and postpartum follow up care for both HIV infected mothers and their infants remains critical to minimize new HIV paediatric infections occurring in the postnatal period.

Overall, the data reported and experiences have been instrumental in shaping global policies, programmatic shifts, investment choices, and to some extent, partnerships. However, additional investments are needed to develop robust routine national monitoring systems that address inequities and disparities and monitor progress towards the Sustainable Development Goals and the target of ending AIDS by 2030.
